# Arthralgia is the main associated symptom to anxio-depressive disorders during the “Long COVID” among Tunisian patients

**DOI:** 10.1192/j.eurpsy.2023.503

**Published:** 2023-07-19

**Authors:** F. Zaouali, A. Chaouch, F. Boubaker, S. Bouchareb, H. E. Mrabet, A. Ben Mabrouk, I. Touil, L. Boussoffara, J. Knani, N. Boudawara, W. Alaya, M. H. Sfar

**Affiliations:** ^1^Endocrinology Depatment; ^2^Pneumology-allergology department, Tahar Sfar University Hospital, Mahdia, Tunisia

## Abstract

**Introduction:**

Various psychiatric disorders were reported during the long COVID. The most frequently cited by physicians included the insomnia, the anxio-depressive disorders and the post-traumatic stress disorder. These symptoms would have a negative impact on the quality of life as well as on the socio-professional and economic efficiency.

**Objectives:**

The aim of this study was to determine the associated factors to anxio-depressive disorders during long COVID.

**Methods:**

A cross sectional analytic study was conducted at Taher Sfar university hospital of Mahdia over a period of one year (from March 2020 to March 2021). It included patients consulting within at least 1 month after a COVID-19 documented infection. We used the Hospital Anxiety and Depression scale (HAD) to screen for anxio-depressive disorders.

**Results:**

We recruited 137 patients in the study. The median age was situated at 60 years, ranging from 17 to 82 years. The sex ratio M/F was 0.073. The median HAD score was 19 [8, 33]. Anxio-depressive disorders were present in 61% of cases. There was no statistically significant association between anxio-depressive disorders and post COVID symptoms except arthralgia and myalgia (38.6% vs 13.5; p=0,006 and 26.8% vs 5.4%; p=0.007, respectively). After the multivariate analysis, only arthralgia during long COVID was associated with the anxio-depressive disorders (95% CI 1.489 to 30.25, p=0.01).

**Conclusions:**

Arthralgia is a frequent symptom sometimes underestimated and in others overtreated. As it seems to be significantly associated with anxio-depressive disorders in the post covid period, physicians should pay attention to the history of a viral documented or probable infection and to psychiatric symptoms’ screening. Our results should however be confirmed by multicenter studies with larger sample size.

**Disclosure of Interest:**

None Declared

